# Combining Co-Amorphous-Based Spray Drying with Inert Carriers to Achieve Improved Bioavailability and Excellent Downstream Manufacturability

**DOI:** 10.3390/pharmaceutics12111063

**Published:** 2020-11-08

**Authors:** Yingxi Zhang, Yuan Gao, Xiaoxiao Du, Rou Guan, Zhonggui He, Hongzhuo Liu

**Affiliations:** Wuya College of Innovation, Shenyang Pharmaceutical University, No.103, Wenhua Road, Shenyang 110016, China; YingxiZhang_SYPHU@163.com (Y.Z.); 15241536560@163.com (Y.G.); duxiaoxiao_dmu@163.com (X.D.); gr_brandy@163.com (R.G.); hezhgui_student@aliyun.com (Z.H.)

**Keywords:** co-amorphous, inert carriers, solubilization, molecular interaction, direct compression

## Abstract

It is crucial to improve poorly water-soluble orally administered drugs through both preclinical and therapeutic drug discovery. A co-amorphous formulation consisting of two low molecular weight (MW) molecules offers a solubility/dissolubility advantage over its crystalline form by maintaining their amorphous status. Here, we report on a co-amorphous solid dispersion (SD) system that includes inert carriers (lactose monohydrate or microcrystalline cellulose) and co-amorphous sacubitril (SAC)-valsartan (VAL) using the spray drying process. The strong molecular interactions between drugs were the driving force for forming robust co-amorphous SDs. Our system provided the highest solubility with more than ~11.5- and 3.12-times solubility increases when compared with the physical mixtures. Co-amorphous lactose monohydrate (LM) SDs showed better bioavailability of APIs (~356.27.8% and 154.01% for the relative bioavailability of LBQ 657 and valsartan, respectively). Co-amorphous inert carrier SDs possessed an excellent compressibility for the production of a direct compression pharmaceutical product. In conclusion, these brand-new co-amorphous SDs could reduce the number of unit processes to produce a final pharmaceutical product for downstream manufacturability.

## 1. Introduction

A large proportion of potential new drugs are classified as poor candidates in clinical trials due to their limited water solubility and unsatisfactory oral bioavailability [1,2]. Even for marketed drugs, the low and variable oral bioavailability of poorly water-soluble drugs remains a major issue for the pharmaceutical industry. To address this problem, several formulation strategies have been developed, including the preparation of pharmaceutical salts [3] or cocrystals [4], pro-drugs [5], and nanomedicines [6]. Amorphous drug delivery systems have been widely demonstrated during the last decade as they provide significantly improved solubility and dissolution performance of active pharmaceutical ingredients (APIs) without the need of chemical modifications, thus, greatly enhancing the oral bioavailability.

Currently, polymer-based amorphous or co-amorphous formulations have been broadly explored in both academic and industrial fields. Co-amorphous formulations consist of two low molecular weight (MW) molecules that stabilize each other in the amorphous form. They have garnered considerable interest in pharmaceutical engineering due to their inherent advantages [7]: firstly, a relatively low amount of co-former is required for the co-amorphous system, and thus an oversized dose unit could be avoided; secondly, hygroscopicity problems with polymeric solid dispersion (SDs) could also be overcome [8]; thirdly, the potential strong molecular interaction between the components could improve the stability of co-amorphous formulation, especially where additional stabilizers were absent [9]. Although the strategy of co-amorphous formulations seems straightforward, the translational development of an effective formulation is challenging. The solubility/dissolution advantage of the co-amorphous systems might be compromised due to the solvent-mediated recrystallization rapidly dissolving from gastrointestinal concentrations above the saturation solubility of APIs are generated [10,11,12].

Various methods have been used to produce co-amorphous systems, such as hot-melt extrusion, solvent evaporation, freeze drying, spray drying, and milling [13]. Among these methods, spray drying was regarded as a reliable large-scale production method for preparing co-amorphous SDs due to its versatility and efficiency [14]. In some cases, spray-dried preparations with a low particle size led to poor powder properties, such as low fluidity and high adhesion, and therefore were extremely difficult to handle [15]. Herein, a third component is typically introduced to improve the properties of the resulting powders by spray drying. Specially, the third component, acting as a carrier excipient, will not be miscible with the co-amorphous component and remains phase-separated from the co-amorphous formation, which allows no interaction with the resulting co-amorphous preparations.

**Figure 1:**
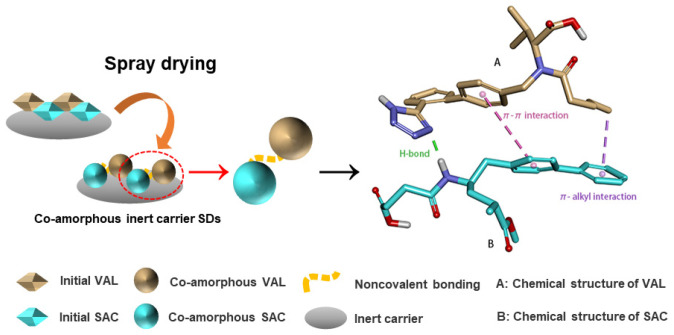
Schematic illustration of the sacubitril-valsartan (SAC-VAL) co-amorphous formulations.

In the current study, a co-amorphous drug/drug combination between sacubitril (SAC) and valsartan (VAL) was chosen to investigate the effect of carrier excipients on the co-amorphous preparations during the spray drying process. The combination of SAC and VAL has demonstrated unprecedented therapeutic effects in the treatment of heart failure [16]. Here, both drugs were transformed into their molecular miscible co-amorphous state, which is desirable in enhancing the oral bioavailability. To avoid the recrystallization risk, it is prudent to involve bulk water unit operations during the process containing co-amorphous systems, such as wet granulation. Accordingly, two diluents/fillers, lactose monohydrate (LM) and microcrystalline cellulose (MCC), were used, which are typical excipients in direct compression formation. The schematic illustration of the SAC-VAL co-amorphous formulations is shown in Figure 1.

The solid state of the preparations was characterized by powder X-ray diffraction (PXRD), Fourier transform infrared spectroscopy (FTIR), and differential scanning calorimetry (DSC). The solubility studies of these co-amorphous systems as well as the supersaturation of both APIs and the simple permeation across artificial membranes were also investigated. Subsequently, an in vivo pharmacokinetic study was performed to assess the impact of co-amorphous SDs on the bioavailability of APIs.

## 2. Materials and Methods

### 2.1. Materials

Valsartan (≥99.9% purity) and sacubitril calcium (≥95.0% purity) were purchased from Shanghai Pharma Group Changzhou Kony Pharmaceutical Co., Ltd. (Changzhou, China) and RuoTai Pharmaceutical Technology Co., Ltd. (Shenyang, China), respectively. Spray-dried lactose monohydrate (LM, FastFlo 316) was kindly supplied by Foremost Farms Co., Ltd. (Kansas, Foremost Farms, USA) and microcrystalline cellulose (MCC, SH-102) was gifted from Sunhere Pharmaceutical Excipients Co., Ltd. (Huainan, Sunhere, China). All chemicals were used as received.

### 2.2. Preparation of Co-Amorphous SDs

Solid particles were prepared using a B-290 mini spray dryer (Buchi, Flawil, Switzerland). SAC and VAL were first weighed into a glass beaker at a 1:1 molar ratio. Ethanol was added to dissolve both components with magnetic stirring. The resulting solution was further adjusted to pH 9 with a sodium hydroxide aqueous solution. To further evaluate the effect of the weight ratio of the inert carrier on the solubility of APIs, SAC and VAL were spray-dried with LM or MCC at the weight ratios (APIs: inert carriers) of 1:1, 1:2, 1:3, and 1:4. The resulting solution or suspensions were delivered using a peristaltic pump at a speed of 10% (3 mL/min) and the aspirator was operated at 40 m^3^/h. The inlet and outlet temperatures were set at 90 °C and 55–65 °C, respectively. A white solid was obtained and then characterized as follows. The spray-dried co-amorphous preparations composition are shown in Table 1.

### 2.3. Characterization of Co-Amorphous SDs

#### 2.3.1. Powder X-ray Diffraction (PXRD) and Fourier Transform Infrared (FT-IR)

The resulting powders were characterized using PXRD and FTIR. PXRD was performed on a DX-2700 machine (HaoYuan Instruments, Dandong, China), which was operated at 45 kV and 40 mA using Cu Kα radiation (λ = 1.54187 Å). The patterns were collected from 3 to 60° (2θ) with a step size of 0.03° (2θ) and a constant counting time of 0.2 s per step.

FTIR was carried out at IFS-55 (Bruker Instruments, Karlsruhe, Germany) by a transmission method using a KBr disk. All spectra were produced by averaging three scans, and the resolution was 1.0 cm^−1^.

#### 2.3.2. Differential Scanning Calorimetry (DSC)

The thermal properties of powders were investigated using DSC. The samples were analyzed on a DSC 2500 (TA Instruments, New Castle, DE, USA) under a nitrogen atmosphere at a flow rate of 20 mL·min^−1^. The substance (~3 mg) was sealed in an aluminum pan, which was subjected to thermal scanning at a heating rate of 20 °C min^−1^. To determine the phase diagram, the samples were heated from 25 °C to 160 °C. To measure the glass transition temperature (*T*_g_), the samples were heated from 25 °C to 160 °C and held isothermally at 160 °C for 1 min. Then, samples were cooled to −20 °C and held isothermally for 1 min following heating to 160 °C.

### 2.4. Particle Size Distribution and Morphology Observations

The geometric particle size distributions (PSDs) of the obtained powders were determined by laser diffraction using a Bettersize 2600 (Bettersize Instruments, Dandong, China). The particles were dispersed using a scirocco dry feeder instrument with 0.3 bar pressure [17].

The surface morphology and particle shape of the samples were captured using a SU8020 scanning electron microscope (SEM, Hitachi, Tokyo, Japan) equipped with a secondary electron detector at 3 kV. The samples were sputter-coated with gold under a vacuum prior to analysis [18].

### 2.5. Apparent Equilibrium Solubility Study

The thermodynamic apparent solubility was measured using an air shaker (QYC-211D, Fuma Laboratory Instruments, Shanghai, China). These samples were kept at 37 ± 0.5 °C with shaking (110 rpm) for 48 h. The suspensions were separated using a GL-16G-II Centrifuge (Anting Scientific Instruments, Shanghai, China) at 13,000 rpm for 5 min. Subsequently, the supernatant was diluted and the concentrations of SAC and VAL were determined using a high-performance liquid chromatography system (Hitachi Instruments, Tokyo, Japan). The drugs were separated using a C_18_ column (250 mm × 4.6 mm, 5 μm. Dikma, Beijing, China). A freshly prepared mixture of acetonitrile and citrate buffer (pH 3.5) (60:40 *v/v*) was used as the mobile phase. The flow rate of the mobile phase was maintained at 1.0 mL/min, and UV detection was set at 255 nm [19].

### 2.6. Dissolution and Permeation Study

#### 2.6.1. No-Sink Dissolution/Permeation (D/P) Study

A dissolution/permeation (D/P) system was used to simultaneously investigate the dissolution and permeation profiles of drugs under the non-sink condition [20,21]. This was utilized for evaluating the ability to prolong the supersaturation of APIs of spray-dried preparations and the artificial membrane permeability of APIs at physiological pH to understand the oral absorption of both drugs. The D/P system (Tianmei Instruments, Shenyang, China) consists of a donor and an acceptor cell with the capacity of 4 mL, and the temperature of the whole system was maintained at 37 °C by a circulating water bath. Both cells were filled with 50 mM phosphate buffer solution (PBS, pH 6.8) and separated by a regenerated cellulose membrane (3500 KDa, Viskase, Chicago, IL, USA). The supersaturated dose (equivalent to 200 mg of co-amorphous API) was examined.

The sample (0.5 mL) was withdrawn from both the donor and acceptor cell at time intervals of 5, 10, 15, 30, 60, 120, and 240 min using a syringe, and any volume change due to the withdrawal was corrected immediately. The concentrations of SAC and VAL in the samples were then determined by high-performance liquid chromatography (HPLC). The existing solid forms were collected at 5 min and 4 h during D/P study. The collected solids were immediately separated from the PBS and dried under room temperature and then were examined by polarized light microscopy (PLM, DM2700P, Leica Microsystems., Wetzlar, Germany). Samples were added onto a glass slide and then covered with a glass coverslip. Images of samples were collected using 20× magnification. After the solubility study, solid residues were immediately filtered, dried, and confirmed by PXRD.

The dissolution performance parameter (DPP) [21] and the flux rate [20] were used to evaluate the dissolubility and permeability of the solid samples.

(1) $$\mathrm{DPP}=\frac{{\mathrm{AUC}}_{C\left(t\right)}-{\mathrm{AUC}}_{C_{R}\left(t\right)}}{{\mathrm{AUC}}_{C_{R}\left(t\right)}}\;\times 100\%$$

where the area under the curve (AUC)_C(t)_ is the amount of drug dissolved and maintained over the period of the dissolution time from 0 to t. The area under the curve AUC_CR(t)_ is the amount of control group, which is the physical mixture (PM) in PBS without a polymer.

The flux rate of a drug through the membrane was calculated using the following equation [20].

(2) $$J\left(t\right)=\frac{\left(C_{t2}-C_{t1}\right)}{A\left(t_{2}-t_{1}\right)}V$$

where *J_(t)_* is the flux rate of the drug; C_t1_ is the drug concentration(mg/mL) at t_1_; C_t2_ is the drug concentration (mg/mL) at t_2_; V is the solution volume, and A is the area of exposed membrane.

#### 2.6.2. Sink Condition Dissolubility Study

To provide insight into the non-supersaturated states of spray-dried preparations, dissolution under sink condition tests were also conducted. The dissolution of the spray drying preparations under sink conditions were also conducted using the United States Pharmacopoeia (USP) paddle method (RC12AD, TDTF Laboratory Instruments, Tianjing, China) with a rotation speed of 50 rpm and 900 mL of phosphate buffer (pH 6.8) was used as a dissolution medium at 37 °C. An equivalent dose of drugs (50 mg co-amorphous APIs) was examined. The samples (5 mL) were withdrawn at a predetermined time point and immediately replaced with 5 mL of buffer solutions. The samples were then determined by HPLC.

### 2.7. Compactibility TEST

To examine how the presence of different excipients affects the bonds between particles during tablet forming, the uniaxial compaction process was recorded using an MPC-100 micro powder characterizer (Okada Seiko, Tokyo, Japan). Die compaction of around 30 mg of spray-dried powders were performed with a 4 mm die, and the platen travel speed was maintained at 0.1 mm/s. The elastic work and plastic work were automatically calculated using the force-displacement profile of the loading and unloading stages of the compaction process under a given compaction stress of 200 kg.

### 2.8. Stability Study

To evaluate the stability, spray-dried preparations (co-amorphous SDs and co-amorphous LM/MCC SDs at 1:3 weight ratio) were stored in desiccators under dry conditions at room temperature for 6 months. They were also stored under accelerated conditions (40 °C/75% RH) in a WD-A drug stability testing instrument (Tianjin Pharmacopoeia Standard Instrument, China) for 1 month. The solid states of the samples were confirmed using PXRD. Chemical stability was tested by dissolving a known amount of the sample and analyzing the sample using HPLC. The additional peaks appearing in the chromatogram were interpreted as degradation products.

### 2.9. Pharmacokinetic Study

#### 2.9.1. Animal Experiments

The animal experimental protocols were approved by the Shenyang Pharmaceutical University Institutional Animal Care and Use Committee (SYPU-IACUC-C2019-8-14-201). The pharmacokinetic study was carried out in male Sprague-Dawley (SD) rats weighing 200–210 g (*n* = 5). Dieting was prohibited for 12 h before the experiment. The tested oral suspensions with equivalent dose (20.57 mg/kg, equivalent to ~200 mg for humans) were prepared by triturating accurately weighed amounts of powdered compounds dispersed in water. After single oral administration, blood was withdrawn at a predetermined time and then, immediately centrifuged at 3500 rpm for 10 min. The resulting plasma samples were stored in polypropylene tubes at −80 °C until further analysis.

#### 2.9.2. Bioanalytical Method

After absorption, SAC is further converted by esterases to the active inhibitor of neprilysin LBQ657 [16]. Therefore, VAL and metabolic product LBQ657 of SAC plasma concentrations were determined using a high-performance liquid chromatography-tandem mass spectrometry (HPLC-MS/MS) method after protein precipitation extraction by using losartan as internal standard. An aliquot of 50 μL IS solution (losartan, 250 ng/mL) was pipetted into a 1.0 mL centrifuge tube. Afterward, 50 μL of plasma and 50 μL of methanol (with 0.1% formic acid) were added. The sample was vortex-mixed for 3 min. Then, 100 μL of methanol (with 0.1% formic acid) was added. The mixture was mixed for 5 min again. After being centrifuged at 13,000 rpm for 10 min, the supernatant liquid was directly injected to the HPLC-MS/MS system.

The chromatography was carried out on an Acquity UPLC^TM^ system (Waters Corp., Milford, MA, USA) with a cooling autosampler. Chromatographic separation was achieved on an ACQUITY UPLC BEH C18 column (50 mm × 2.1 mm, 2.6 μm). The mobile phase A consisted of methanol with 0.1% formic acid, and mobile phase B was composed of 5 mmol/L ammonium acetate. The flow rate was set at 0.2 mL/min. The gradient elution was performed: 0–1.0 min, 20% A; 1.0–2.2 min, 80% A; 2.2–2.5 min, 20% A; and 2.5–4 min, 20% A. The injection volume was 5 μL.

Mass spectrometric detection was performed on a tandem quadrupole detector equipped with an electron spray ionization (ESI) source. The ESI source was set in positive mode. The two compounds were detected using multiple reaction monitoring (MRM) of the transition of m/z 384→366.1 for LBQ657, 458.24→300.02 for VAL, and 423.2→207.1 for IS. The optimal MS parameters were set as follows. The capillary voltage was 3.2 kV, the cone voltage was 40 V for LBQ657, 40 V for VAL, and 28 V for IS; the source temperature was kept at 120 °C; and the desolvation temperature was kept at 500 °C. The optimized collision energy was 22 eV for LBQ657, 20 eV for VAL, and 18 eV for IS. Nitrogen was used as the desolvation and cone gas with flow rates of 650 and 60 L/h, respectively.

All data collected were examined using the Masslynx^TM^ NT 4.1 software with the QuanLynx^TM^ program (Waters Corp., Milford, MA, USA). The pharmacokinetic parameters were computed using DAS 2.0 software with a statistical moment model.

### 2.10. Statistical Analyses

Statistical analysis was performed using one-way ANOVA by SPSS statistics 25, and the level of significance was accepted at *p* < 0.05.

## 3. Results

### 3.1. Solid State Characterization

**Figure 2:**
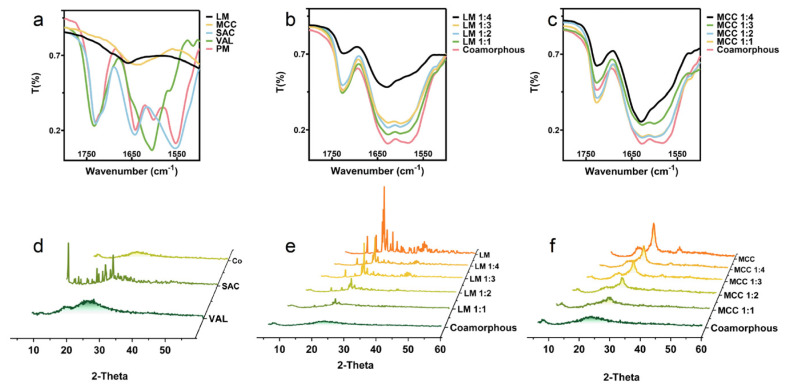
Characterization of spray-dried amorphous SDs, single drugs, and inert carriers. (**a**–**c**) Fourier transform infrared spectroscopy (FTIR) spectroscopy; (**d**–**f**) powder X-ray diffraction patterns.

To investigate the possible intermolecular interactions between both drugs and drug-carriers, FTIR spectroscopy analysis was initially conducted upon the resulting powders [22]. Since LM and MCC show broad O-H groups stretching vibrations that usually overlap the spectra of N-H group stretching vibrations of APIs, which appear at 3500–3100 cm^−1^, this band is not discussed in the article [23]. Figure 2 shows the collected spectra in the region from 1500 to 1800 cm^−1^ (The whole patterns are shown in Figure S1). As shown in Figure 2a, SAC displays stretching vibration of an amide C=O group (amide I) at 1642.9 cm^−1^, combination (amide II) of the C−N stretching, N−H bending at 1554.0 cm^−1^, and the stretching vibration of a carboxylate C=O group at 1729.4 cm^−1^. The amide C=O group (amide I) of VAL shows a stretching vibration at 1605.4 cm^−1^ and carboxylic acid C=O group at 1733.3 cm^−1^, which are in good agreement with those results mentioned in the literature [24].

In co-amorphous samples, the amide bands of APIs overlap together, which can be seen clearly in Figure 2b. Any shift of the amide frequency for VAL is covered up. By comparing co-amorphous formulations to the physical mixture, we found hydrogen-bonding interaction between SAC and VAL, by red shifting of the amide I frequency (1642.9→1627.5 cm^−1^) and blue shifting of the amide II frequencies (1554.0→1582.7 cm^−1^) of SAC [25]. In addition, the SAC carboxylate C=O group stretching vibration bands shows red shifts from 1729.4 cm^−1^ toward 1725.8 cm^−1^, indicating another hydrogen-bonding site. The missing carboxylic acid C=O group vibrational band (1733.3 cm^−1^) of VAL is mainly ascribed to form carboxylates [26].

Figure 2d,e shows the PXRD patterns of SAC, VAL, and the spray-dried preparations. As shown in Figure 2d, the patterns for pure SAC and VAL show characteristic peaks and a typical halo in the diffractograms, indicating crystalline and amorphous forms, respectively. A halo was also observed in the spray-dried SAC-VAL, suggesting an amorphous state [27]. When LM is used as the carrier, the characteristic diffraction peaks of LM remain in the spray-dried preparations, and the peak intensity gradually increases with the increase of the LM weight ratio (Figure 2e) [28].

Notably, there is no evidence of characteristic peaks ascribed to SAC observed in the above preparations, which indicates the amorphous state of SAC. In the case of the original MCC, there are three main reflections at 2*θ* of 15.67°, 22.74°, and 34.73°, attributed to 60–70% of the crystalline (Figure 2f) [29]. In line with the preparations containing LM, the spray-dried powders show the characterized peaks of MCC with the absence of the peaks of SAC, confirming the amorphous state of API. Combined with the FTIR and PXRD data, we, therefore, concluded that co-amorphous powder of APIs were obtained after the spray-drying process.

**Figure 3:**
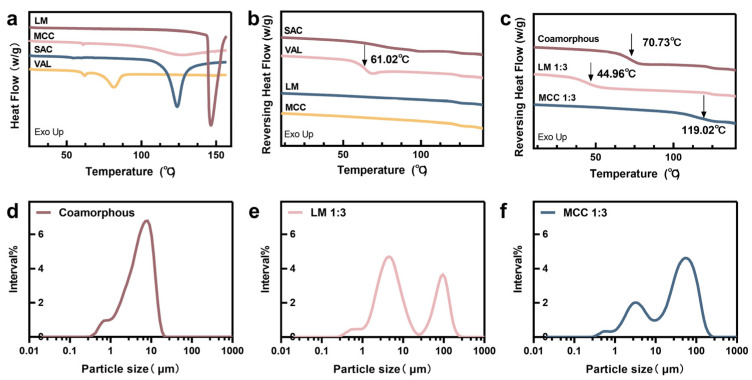
(**a**–**c**) Representative differential scanning calorimetry (DSC) thermograms of spray-dried samples, single drug, and inert carriers. The *T*_g_ are indicated by black arrows. (**d**–**f**) Particle size distribution curves of representative preparations.

To better understand the effect of inert carriers on co-amorphous SDs, the co-amorphous SDs at the weight ratio of 1:3 between APIs and LM or MCC were analyzed using DSC. The conventional DSC thermogram is shown in Figure 3a. SAC shows a sharp melting endothermic temperature (*T*_m_) at 123.14 °C. VAL gives two endothermic peaks at approximately 62.01 °C (a small broad peak) and 81.48 °C (a broad peak), due to water evaporation and a glass transition with high enthalpy relaxation peak overlapped with a change of heat capacity [30]. The glass transition temperature (*T*_g_) of single VAL appears at 61.02 °C as shown in the second step heating curve (Figure 3b). Specifically, a single glass transition peak at 70.73 °C is observed for spray-dried SAC-VAL in Figure 3c, representing the formed homogeneous phase after spray-drying.

SAC acts as an anti-plasticizer as the *T*_g_ of the resulting preparation is higher than that of single VAL [14]. Co-amorphous LM SDs show a lower *T*_g_ (~44.96 °C) as compared with the preparation without the carrier, while a sharp melting endotherm of LM is also maintained in the conventional DSC thermogram (Figure S3). Thus, the SAC and VAL in the mixture were completely dissolved in each other as a co-amorphous state, but were not miscible with LM. The decreased *T*_g_ might be due to the increased molecular mobility of the SD systems [31]. On the contrary, an increased *T*_g_ (~119.02 °C) is observed in the thermogram of co-amorphous MCC SDs, indicating higher stability.

### 3.2. Particle Size and Morphology

**Figure 4:**
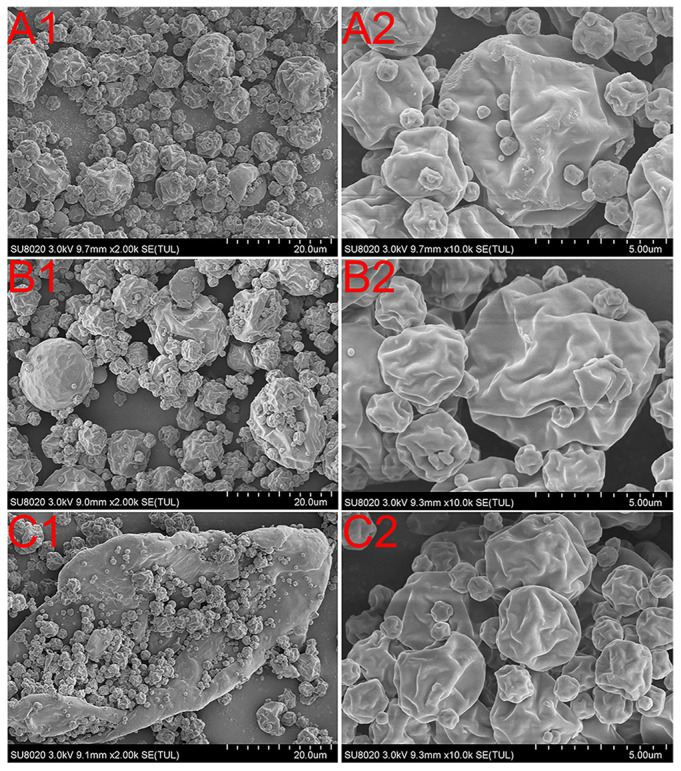
Scanning electron micrographs of (**A1**,**A2**) co-amorphous SDs (**B1**,**B2**) co-amorphous LM SDs at weight ratio 1:3 (**C1**,**C2**) co-amorphous MCC SDs at a weight ratio of 1:3.

A dry powder laser particle size analyzer was used to analyze the particle distribution of the spray-dried preparations. The powder of co-amorphous SAL-VAL had a nearly single size distribution with the D_50_ of 5.09 μm (Figure 3d). Indeed, the size distribution of the powder of SDs with carriers shows two different populations (Figure 3e,f), one for co-amorphous particles (D_50_ ~5.65 μm and ~4.02 μm for the samples with LM or MCC, respectively) and another one for the inert excipient. To confirm this supposition, the morphology of the corresponding sample was then investigated using SEM. As shown in Figure 4, the particles of co-amorphous APIs (1–10 µm) are nearly spherically shaped with a wrinkled surface. The solvent underwent flash atomization rather than pressure-induced atomization in the high temperature during the spray-drying, which led to the collapsed morphology of particles [32]. The fine particles were observed in co-amorphous LM SDs in addition to the wrinkled particles, indicating that the aggregation of LM was associated with co-amorphous particles of APIs. Similarly, there was a population of large plate-like particles with the backpack of many co-amorphous particles in co-amorphous MCC SDs.

### 3.3. Apparent Equilibrium Solubility

**Figure 5:**
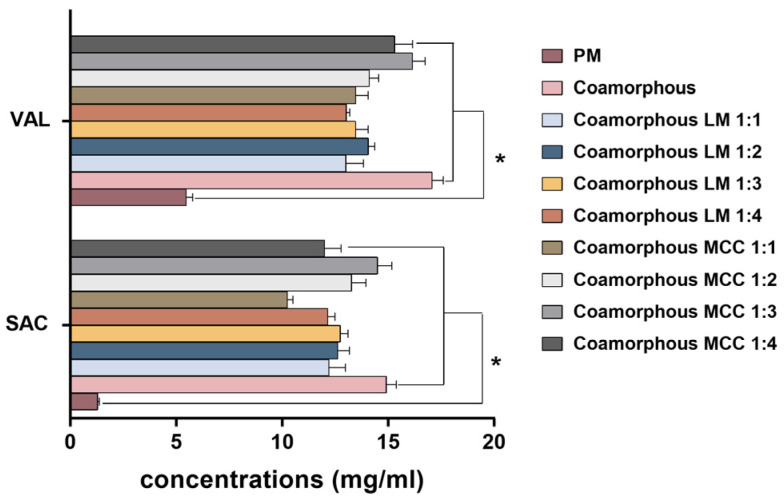
The equilibrium solubility of spray-dried preparations and physical mixtures (PM) in 50 mM pH 6.8 PBS (*n* = 3). Statistical analysis was determined using one-way ANOVA (*: *p* < 0.05).

Various strategies have been designed for improving the solubility of poorly water-soluble drugs since the rate-limiting step of oral absorption is dominated by the kinetics of dissolution of the APIs from the solid forms [33]. To assess the co-amorphous solubility advantages, the solubilities of both APIs and the spray-dried preparations in PBS 6.8 were determined and the results are outlined in Figure 5. Briefly, all of the spray-dried preparations significantly increased the solubility of APIs when compared with physical mixtures. In particular, co-amorphous SAC-VAL provided the highest solubility (14.92 mg/mL for SAC and 17.07 mg/mL for VAL), which were ~11.5 and ~3.1 times increases compared with the physical mixtures (1.29 mg/mL for SAC and 5.47 mg/mL for VAL), respectively. When LM and MCC were used as the carriers, there was a slight decrease in the solubility of SAC and VAL as compared with the co-amorphous SAC-VAL; however, the solubilities were remarkably higher than those of the physical mixture.

There was a slight difference in the solubility of SAC and VAL in the co-amorphous LM SDs at various weight ratios, where the co-amorphous LM SDs at a 1:3 weight ratio appeared to be higher than the others for SAC (12.15 mg/mL), and at a 1:2 weight ratio for VAL (14.06 mg/mL). In contrast, the differences of solubilities between the co-amorphous MCC SDs at different weight ratios were observed, where co-amorphous MCC SDs at a 1:3 weight ratio possessed significant superiority (14.50 mg/mL of SAC and 16.16 mg/mL of VAL).

### 3.4. Dissolution and Permeation Behavior

**Figure 6:**
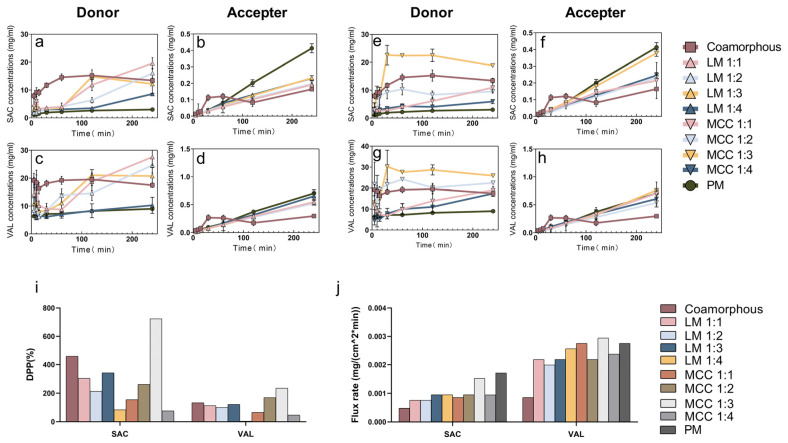
The dissolution and permeation properties of physical mixture (PM), co-amorphous SDs, (**a**–**d**) co-amorphous LM SDs and (**e**–**h**) co-amorphous MCC SDs in PBS (*n* = 3). (i) DPP comparison of co-amorphous SDs and co-amorphous inert carriers SDs. (**j**) The flux rate of physical mixture (PM), and spray-dried preparations.

The thermodynamically unstable amorphous preparations might induce phase inversion to crystalline forms once dissolved in water, which limits their dissolution advantages [34]. To quantify the effect of inert carriers on the co-amorphous APIs, particularly in the evaluation of their ability to prolong the supersaturation of APIs, the DPP parameters in different supersaturated systems were calculated and evaluated by D/P systems [21]. By the same D/P systems, the artificial membrane permeability of APIs at physiological pH was also assessed to determine oral absorption of both drugs. The dissolution and permeation properties of APIs in LM- and MCC-containing preparations are shown in Figure 6a–d and Figure 6e–h, respectively.

For co-amorphous APIs without carriers, we observed that the dissolved SAC gradually increased from 7.92 mg/mL at 5 min to their maximum concentrations (15.22 mg/mL) at 120 min and then slightly decreased (Figure 6a). The amount of VAL initially reached the maximum concentrations (19.18 mg/mL) and rapidly decreased to minimum concentrations (16.40 mg/mL) at 15 min followed by slow further increases (Figure 6c). However, co-amorphous SAC-VAL with inert carrier systems exhibited distinct dissolution profiles.

In general, for co-amorphous LM SDs, both APIs started at a lower concentration and then, gradually increased to their maximum concentrations. Conversely, both APIs dissolved from MCC SDs rapidly. All the spray-dried SDs demonstrated enhanced dissolution properties in comparison to their physical mixtures, except for the co-amorphous LM SDs at the 1:4 weight ratio. Figure 6i demonstrates the dissolution properties and supersaturated state of APIs in the donor compartment in comparison to the physical mixtures of SAC and VAL.

Notably, co-amorphous APIs without any carriers significantly improved the drug dissolution as the DPP was 459.09% higher than that of physical mixtures for SAC and was 133.28% higher for VAL. For the co-amorphous LM SDs, the highest improvement in the supersaturated state was observed at the 1:3 weight ratio as the DPP of SAC and VAL were promoted to be 342.28% and 121.03% compared with those in physical mixtures. In the case of MCC SDs, the system at the 1:3 weight ratio of APIs and carriers exhibited an optimal improvement in the supersaturated performance compared with other weight ratios as evidenced with a 724.19% DPP of SAC and 235.62% DPP of VAL (*p* < 0.05).

The permeabilities of APIs were observed for all of the preparations and the flux rates were calculated (Figure 6j). Unexpectedly, co-amorphous systems impaired the permeation of APIs compared with the physical mixtures, where the flux rate decreased by 27.78% for SAC and 31.03% for VAL. This is not surprising as a viscous soft mass was observed to adhere upon the wall of the donor cell near the regenerated cellulose membrane. Normally, co-amorphous systems possess a high hydration ability to trap water molecules via capillary forces and surface tension [35]. Once the diffusion rate of water is much higher than that of APIs, a soft mass occurred and finally led to the decreased flux. On the other hand, as compared with physical mixtures, the relatively poor permeability of co-amorphous systems indicated that strong intermolecular interactions remained in the aqueous environment.

To provide insight into the different supersaturated states of spray-dried preparations, dissolution under sink condition tests were also conducted (Figure S2). Dissolved amounts of spray-dried preparations reached 100% in 5 min indicating that co-amorphous spray-dried preparations gained a satisfactory dissolution rate in spite of the different supersaturated states. As the optimal improvement in the supersaturated state was observed at the 1:3 weight ratio of API and carriers, these systems were chosen for the following studies.

PLM images of the solid residuals retrieved from the above D/P tests are shown in Figure S4. SAC-VAL co-amorphous transformed to aggregates of needle-like crystals within 5 min. However, the amount of needle-like crystals decreased significantly at the presence of LM/MCC. Key characteristic peaks of SAC-VAL cocrystal were found at 2θ = 4.20°, 4.98°, 5.39°, 5.44°, 12.63° (Cambridge Structural Database card, No. 72-6902). The new needle-like crystals were proved to be the SAC-VAL cocrystals by the unique features of SAC-VAL cocrystal by the diffraction peaks at 12.63° (2θ) in the PXRD pattern. The intensity of the characteristic peak decreased significantly with the presence of inert carriers in preparations. The PXRD data suggested that the phase transformation was delayed when LM/MCC was present (Figure S5). The abnormal APIs release rates in different spray-dried samples, indicating the change of properties of solids.

### 3.5. Compactibility Test

**Figure 7:**
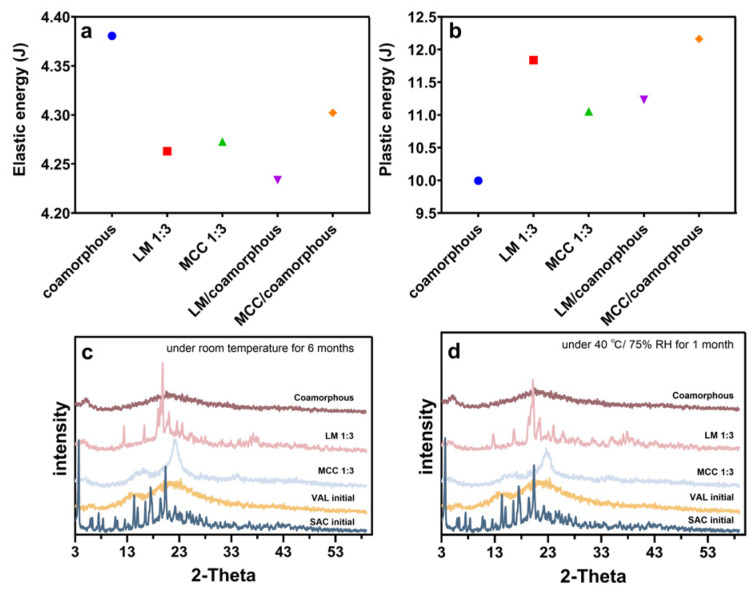
Elastic work (**a**) and plastic work (**b**) in joules on co-amorphous and co-amorphous LM or MCC SDs at a 1:3 weight ratio and binary mixture of co-amorphous and inert carriers at corresponding weight ratios. Powder X-ray diffraction (PXRD) patterns of spray-dried preparations (**c**) under room temperature for 6 months, (**d**) under 40 °C/75% RH for 1 month.

The feasibility of co-amorphous inert carrier SDs to produce a final direct compression pharmaceutical product was evaluated by compaction studies. Figure 7a,b shows the plastic work and elastic work of co-amorphous SAC-VAL, co-amorphous inert carriers SDs, physical mixtures of co-amorphous SAC-VAL, and the corresponding carriers. We observed the highest increase in the plastic energy of co-amorphous LM SDs at the 1:3 weight ratio when compared to the other samples. On the other hand, the plastic energy for co-amorphous MCC SDs was less than their simple mixture, but still larger than for co-amorphous SAC-VAL. Hence, co-amorphous inert carrier SDs can allow for removal of the blending step, going directly from a spray-dried process to a direct compression.

### 3.6. Stability Study

The preliminary stability of spray-dried preparations was tested to explore the future development potential. Figure 7c shows the storage stabilities at room temperature for six months, while samples in Figure 7d were stored at 40 °C/75% RH for 1 month. The PXRD pattern of SAC had sharp peaks at 3.7°, 14.6°, 15.3°, 16.8°, 17.9°, 19.7°, 20.7°, and 22.6°; and diffraction angles (2θ), which represented the typical crystalline form of the drug. The PXRD pattern of the SAC-VAL cocrystal (Cambridge Structural Database card, No. 72-6902) had sharp peaks at 4.20°, 4.98°, 5.39°, 5.44°, and 12.63°. Co-amorphous SAC-VAL and LM/MCC-based SDs remained in an amorphous state without any obvious crystal peaks under the experimental process. Therefore, these spray-dried samples showed high physical stability. The chemical stability was confirmed by HPLC analysis. No novel impurities were found for any of the samples after storage at any conditions (Figure S6).

### 3.7. Pharmacokinetic Studies

**Figure 8:**
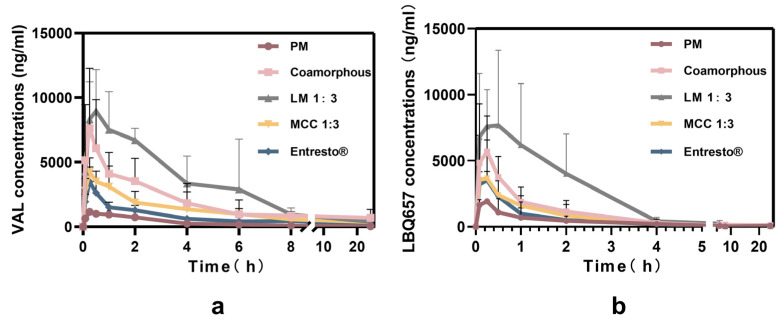
The mean plasma concentrations vs. time profiles of (**a**) VAL and (**b**) LBQ657 after single oral administration to SD rats (*n* = 5).

The oral bioavailability of spray-dried preparations was then carried out in SD rats with the marketed formulation (Entresto^®^) as a control. As SAC was further rapidly converted to the active neprilysin inhibitor LBQ657 in rat plasma, the concentrations of VAL and LBQ657 were selected as the measured parameters. The profiles of drug concentrations in plasma versus time are presented in Figure 8. The pharmacokinetic parameters are shown in Table 2.

Overall, the spray-dried preparations provided enhanced plasma concentrations compared with those of Entresto^®^ and the physical mixture over 24 h. Due to the low solubility of APIs, physical mixture showed the lowest plasma concentrations. Furthermore, according to the AUC_0-t_ values, the relative bioavailability of different preparations compared with the marketed formulation have been calculated and listed in Table 2. LM SDs gave significantly higher initial plasma concentrations than other preparations. In particular, the AUC_0-t_ values of LM SDs were ~1.54- (VAL) and ~3.56- (LBQ657) folds higher than that of Entresto^®^, indicating LM SDs could effectively increase the relative bioavailability compared with the commercial product. The MCC SDs displayed similar relative bioavailability as the Entresto^®^, ~1.19- (VAL) and ~1.25- (LBQ657) fold. Co-amorphous APIs resulted in a minor improvement at ~1.39- (VAL) and ~2.31- (LBQ657) folds higher than Entresto^®^. Hence, our results suggest that LM SDs have a good application prospect for SAC-VAL clinical application.

## 4. Discussion

Co-amorphous systems were formulated with APIs or in combinations with inert carriers by spray drying in the present study. Although the carriers are not involved in the formation of co-amorphous systems, they can influence the dissolution, permeation, and, thus, oral absorption of APIs.

Spray drying is a widely used method to produce particles by transformation of a fluid material into dried particles [36,37,38]. The fast evaporation of the solvent during the drying process typically results in predominately amorphous material for small molecular substances [36]. Such processes, however, were shown to produce too small SAC-VAL dried microparticles with poor flow ability, and APIs needed to be co-spray-dried with some excipients to obtain desirable pharmaceutical powders [39]. LM and MCC as inert carriers were introduced in spray-dried systems to improve the powder properties of dried particles. The molecular interaction between APIs and the excipients were investigated using FTIR, PXRD, and DSC in the present study.

The molecular interactions through hydrogen bond formation between SAC and VAL spray-dried co-amorphous were characterized by: (i) a red shift in the amide I frequency (1642.9→1627.5 cm^−1^) and a blue shift in the amide II frequencies (1554.0→1582.7 cm^−1^) of SAC; (ii) a red shift in the carboxylate C=O group stretching vibration bands of SAC from 1729.4 cm^−1^ toward 1725.8 cm^−1^. The same bands positions were observed for both the spray-dried co-amorphous and the spray-dried co-amorphous inert carrier SDs (containing LM or MCC). However, there were only weaker corresponding bands but no new ones observed when LM or MCC were used, indicating that the excipients had less interaction on the hydrogen bonds between SAC and VAL (Figure 2a–c). The result was confirmed by PXRD, where only co-amorphous halos as well as the peaks of LM or MCC were observed, likely due to no interaction between the co-amorphous components and the carriers, as previously commented.

The single *T*_g_ indicates SAC and VAL in the co-amorphous state were completely dissolved in each other as a homogeneous phase [40]. The co-amorphous LM SDs showed *T*_g_ values that were lower than the corresponding values of co-amorphous and co-amorphous MCC SDs (Figure 3c). This indicates that the added LM might increase the molecular mobility of the solid dispersion systems and that MCC enhances the stability of co-amorphous SDs. DSC thermograms also showed the melting temperature of LM and the *T*_g_ of co-amorphous in co-amorphous LM SDs at the 1:3 weight ratio (Figure S3), indicating that the co-amorphous phase remained separated from LM. On the other hand, the melting temperature of MCC was not recorded within the investigated temperature range since the melting point of MCC is higher than the decomposition temperature of VAL.

The supersaturated state remained in most of the investigated systems, attributed to the strong molecular interactions between APIs. Specifically, a qualitative order of dissolubility might be stated as follows: co-amorphous MCC SDs > co-amorphous SAC-VAL > co-amorphous LM SDs (Figure 7i). As depicted, a viscous soft mass was observed when an overdose of spray-dried preparations was added into the donor cell, which hindered the wettability of APIs. When LM was used as a carrier, competitive interactions with water occurred and led to worse wettability of APIs and, then, poor initial dissolution as compared with co-amorphous SAC-VAL. Conversely, a less viscous soft mass was observed in the case of MCC SDs, which allowed the favorable dispersion of APIs particles and eventually resulted in better dissolubility. SAC-VAL cocrystals were formed in the solid residues within 5 min during no-sink condition dissolution/permeation study. The intensity of characteristic peak decreased significantly with the presence of inert carriers in preparations, suggesting the phase transformation was delayed when LM/MCC was present (Figures S4 and S5). Notably, no viscous mass was observed in the sink condition tests as abundant water quickly wetted the prepared powders, leading to very fast dissolution. Despite suffering recrystallization exposure to no-sink condition, co-amorphous SDs offered rapid drug dissolution rates and formed drug supersaturated solutions. Spray-dried preparations improved the oral bioavailability of APIs as compared with the physical mixture and the market preparations (Figure 8). Unexpectedly, the co-amorphous LM SDs resulted in excellent bioavailability in vivo (~356.27.8% and 154.01% for the relative bioavailability of LBQ 657 and VAL, respectively) when the commercial product was used as a control. The possible reasons for these discrepancies are discussed below.

It has been suggested that molecular interactions between excipients and co-amorphous molecular could strongly alter the drug dissolution rate, even for dissolution mechanism [41,42]. An insightful understanding of interactions between the co-amorphous and LM/MCC would be required to further elucidate the different effects of inert carriers on bioavailability of each of the co-amorphous SDs. It is currently challenging to experimentally measure this interaction. The molecular interactions between drugs and carrier in solution can been investigated through molecular dynamics simulation approaches to reveal the mechanisms of drugs dissolution [43]. Compared with MCC, LM has more hydrogen bond donors, which can influence the supramolecular structure of SAC-VAL in solution phase.

For orally administered drugs, a satisfactory solubility as well as a sufficient permeability from membranes of the gastrointestinal (GI) tract is the essential prerequisite for sufficient systemic absorption [44]. It is assumed that SAC-VAL-LM can exist as ternary molecular aggregates in solution phase, which can modify membranes permeability of the GI tract. The different drug−carrier hydrogen bonding interactions in solution phase might be translated to unequable bioavailability. Therefore, a membrane permeability study in the well-established epithelial cellular models will be investigated in our further research. Furthermore, utilizing fast-dissolving excipients as carriers is an effective way to enhance drug dissolution [45]. The solubility of LM in water was significantly higher than that of MCC, which might result in higher bioavailability in vivo. The pharmacokinetic study was carried out in SD rats by single oral suspensions where the degree of supersaturation was much lower than the D/P test, resulting in a poor in vitro–in vivo relationship. We speculate that a better in vitro–in vivo relationship could be obtained by using tablets in dissolubility and bioavailability studies. In spite of this, spray-dried co-amorphous LM SDs were exhibited to be a promising oral drug delivery system for SAC and VAL. Co-amorphous SDs and co-amorphous inert carrier SDs were stable for up to six months at room temperature and 1 month at 40 °C/75% RH. As co-amorphous inert carrier SDs possessed superior compression properties, this indicates excellent downstream manufacturability.

## 5. Conclusions

This study demonstrated the feasibility of SAC-VAL co-amorphous formulations, in particular with the inclusion of inert carriers by spray drying. Co-spray drying LM or MCC and the co-amorphous components provided improved powder properties of co-amorphous APIs. The ratio of excipients and APIs was optimized based on the supersaturated state and permeation in vitro. Finally, as well as influencing the dissolution, permeation, and, thus, oral absorption of APIs, co-amorphous LM SDs led to excellent bioavailability of APIs (~356.27.8% and 154.01% for the relative bioavailability of LBQ 657 and valsartan, respectively) when compared with the physical mixture of APIs.

## Figures and Tables

**Table 1:** Spray-dried co-amorphous solid dispersion (SD) preparation compositions. Lactose monohydrate (LM), and microcrystalline cellulose (MCC).

Formulation	SAC (g)	VAL (g)	LM (g)	Formulation	SAC (g)	VAL (g)	MCC (g)
Co-amorphous	1.33	1.34	-				
Co-amorphous LM 1:1	1.33	1.34	2.67	Co-amorphous MCC 1:1	1.33	1.34	2.67
Co-amorphous LM 1:2	1.33	1.34	5.34	Co-amorphous MCC 1:2	1.33	1.34	5.34
Co-amorphous LM 1:3	1.33	1.34	8.01	Co-amorphous MCC 1:3	1.33	1.34	8.01
Co-amorphous LM 1:4	1.33	1.34	10.68	Co-amorphous MCC 1:4	1.33	1.34	10.68

**Table 2:** Pharmacokinetic data of VAL and LBQ657 (*n* = 5, mean ± SD).

**Parameters**	**VAL**				
	**PM**	**Co-amorphous**	**LM 1:3**	**MCC 1:3**	**Entresto**
AUC_(0-_t)__(ng h/mL)	3574.74 ± 933.15	57,022.08 ± 833.40	63,013.11 ± 23,471.31	48,748.19 ± 53,022.52	40,916.24 ± 19,884.30
C_max_ (ng/mL)	1175.18 ± 220.28	11,352.98± 4598.69	7699.03 ± 3200.25	6163.12 ± 1599.95	5289.11 ± 1851.86
Relative bioavailability %	10.63	139.36	154.01	119.14	-
**Parameters**	**LBQ657**				
	**PM**	**Co-amorphous**	**LM 1:3**	**MCC 1:3**	**Entresto**
AUC_(0-_t)__(ng h/mL)	3625.72 ± 1716.67	13,258.26 ± 1325.30	20,454.46 ± 7323.42	7159.44 ± 18,014.82	5741.35 ± 1137.78
C_max_ (ng/mL)	3074.73 ± 949.17	6363.13 ± 2706.14	11,243.79 ± 3936.34	4134.42 ± 624.32	3841.42 ± 3473.21
Relative bioavailability %	63.15	230.93	356.27	124.70	-

## Supplementary Materials

The following are available online at https://www.mdpi.com/1999-4923/12/11/1063/s1, Figure S1: The whole FTIR patterns of spray-dried samples, Figure S2: Dissolution profiles of spray-dried samples under sink conditions. (*n* = 3), Figure S3: DSC thermograms for co-amorphous LM SDs. Ramp 20 °C/min to 160.00 °C (blue curve); Isothermal 1.0 min; Ramp 20 °C/min to −20.00 °C (red curve); Isothermal 1.0 min; Ramp 20 °C/min to 160.00 °C (purple curve), Figure S4: PLM images of spray-dried samples collected at 5 min and 4 h during D/P study. Figure S5: PXRD patterns of solid residues after D/P study. Figure S6: Liquid chromatogram of spray-dried samples (a–c) under room temperature for 6 months, (d–f) under 40 °C/75% RH for 1 month.
