# Supplementary material for: Combining Co-Amorphous-Based Spray Drying with Inert Carriers to Achieve Improved Bioavailability and Excellent Downstream Manufacturability
Source: Pharmaceutics. 2020 Nov 8;12(11):1063. doi: 10.3390/pharmaceutics12111063 (PMC7695141; doi:10.3390/pharmaceutics12111063)
Supplement: Supplementary file 1 [file pharmaceutics-12-01063-s001.pdf]

pharmaceutics

Supplementary Materials: Combining Co-Amorphous-Based Spray Drying with Inert Carriers to Achieve Improved Bioavailability and Excellent Downstream Manufacturability

Yingxi Zhang, Yuan Gao, Xiaoxiao Du, Rou Guan, Zhonggui He and Hongzhuo Liu *

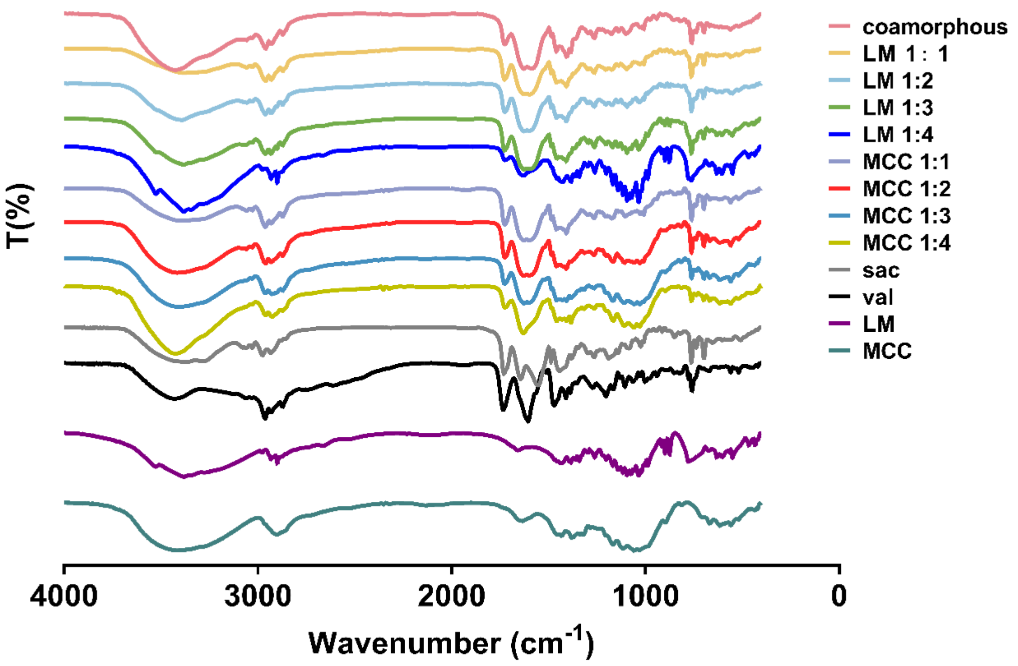

Figure S1. The whole FTIR patterns of samples.

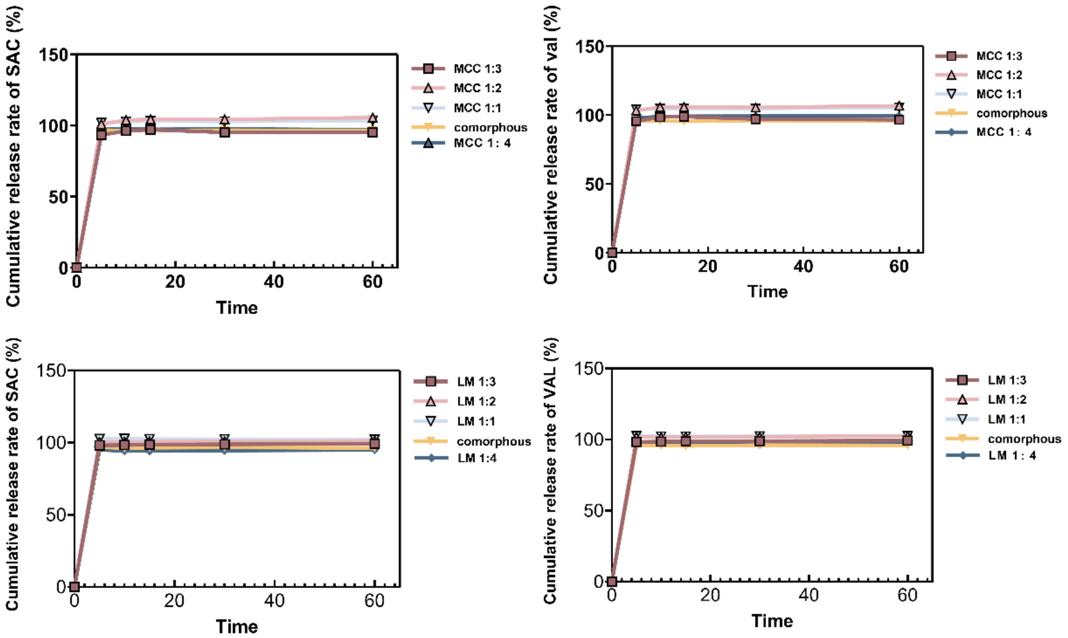

Figure S2. Dissolution profiles of spray-dried samples under sink condition. (n=3).

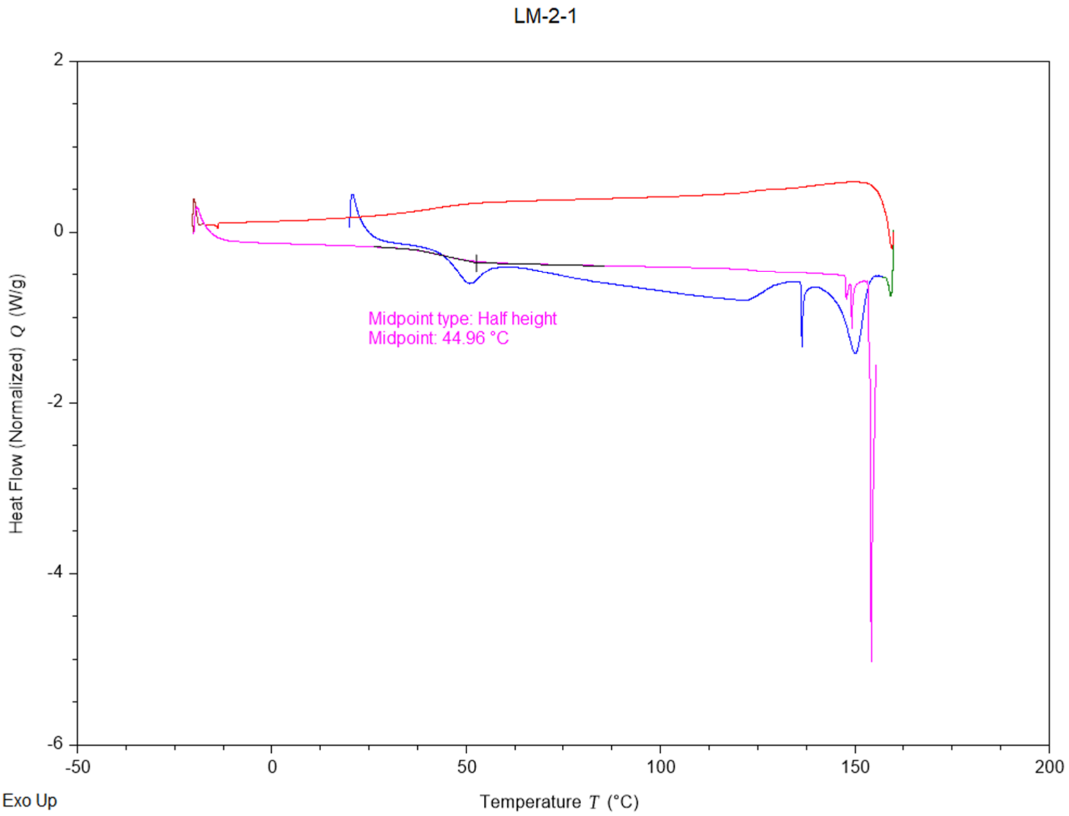

Figure S3. DSC thermograms for coamorphous LM SDs. Ramp 20 °C/min to 160.00 °C (blue curve); Isothermal 1.0 min; Ramp 20 °C/min to -20.00 °C (red curve); Isothermal 1.0 min; Ramp 20 °C/min to 160.00 °C (purple curve).

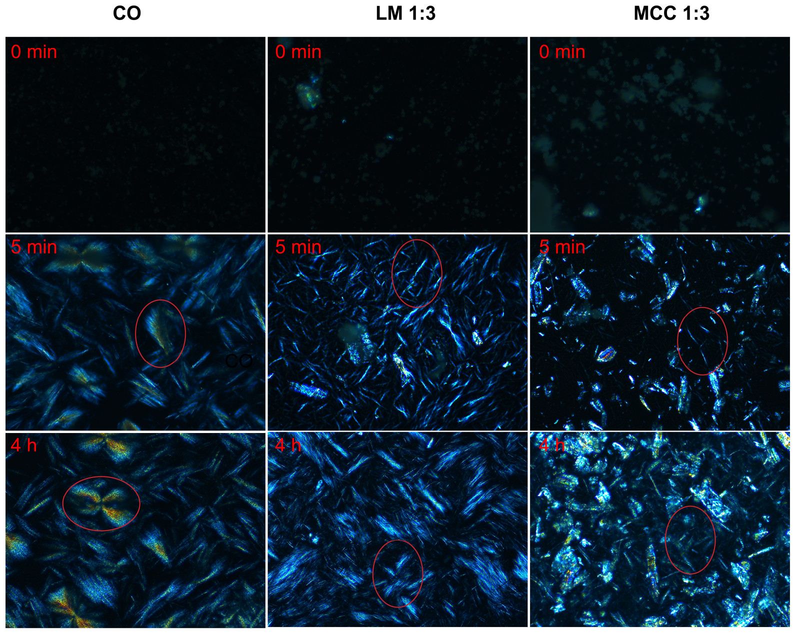

Figure S4. PLM images of spray-dried samples collected at 5 min and 4 h during D/P study.

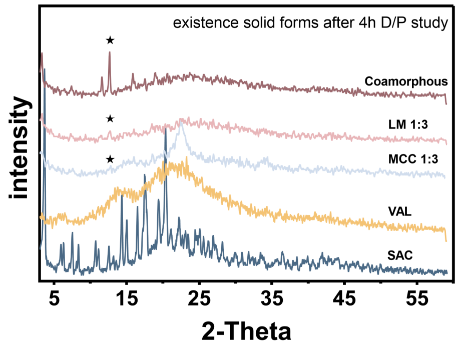

Figure S5. PXRD patterns of solid residues after D/P study.

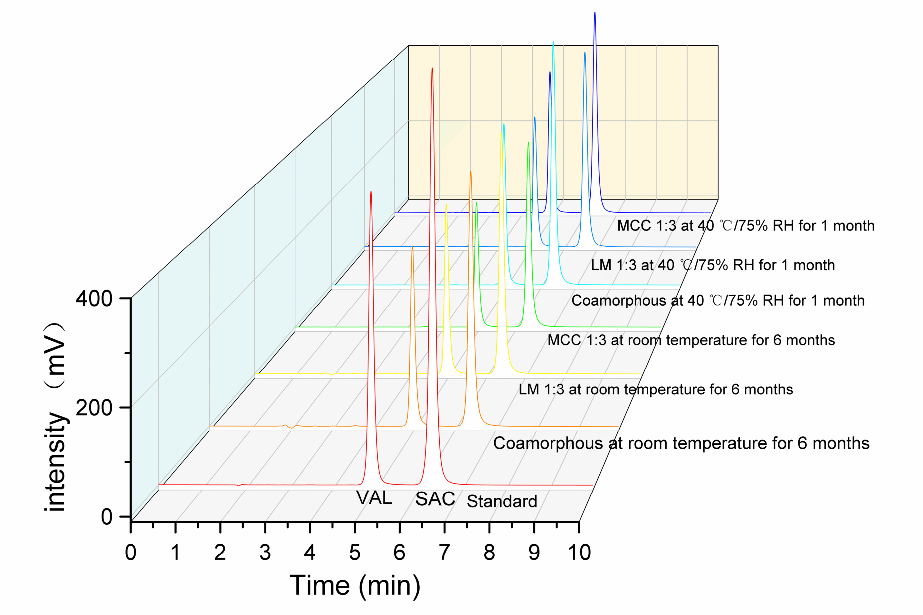

Figure S6. Liquid chromatogram of spray-dried samples under room temperature for 6 months and under 40 ℃/ 75% RH for 1 month. .
